# Comparison of low-concentration carbon dioxide-enriched and tap water immersion on body temperature after passive heating

**DOI:** 10.1186/s40101-021-00271-z

**Published:** 2021-11-17

**Authors:** Keiji Hayashi

**Affiliations:** Junior College, University of Shizuoka, 2-2-1 Oshika, Suruga-ku, Shizuoka, 422-8021 Japan

**Keywords:** CO_2_-enriched water, Body temperature, Cutaneous vasodilation, Cooling, water immersion

## Abstract

**Background:**

Because carbon dioxide (CO_2_)-enriched water causes cutaneous vasodilation, immersion in CO_2_-enriched water facilitates heat transfer from the body to the water or from the water to the body. Consequently, immersion in CO_2_-enriched water raises or reduces body temperature faster than immersion in fresh water. However, it takes time to dissolve CO_2_ in tap water and because the dissolved CO_2_ concentration decreases over time, the actual CO_2_ concentration is likely lower than the stated target concentration. However, it is unclear whether water containing a lower CO_2_ concentration would also cool the body faster than fresh water after body temperature had been increased.

**Methods:**

Ten healthy males (mean age = 20 ± 1 years) participated in the study. Participants were first immersed for 15 min in a tap water bath at 40 °C to raise body temperature. They then moved to a tap water or CO_2_-enriched water bath at 30 °C to reduce body temperature. The CO_2_ concentration was set at 500 ppm. The present study measured cooling time and cooling rate (slope of the regression line relating auditory canal temperature (*T*_ac_) to cooling time) to assess the cooling effect of CO_2_-enriched water immersion.

**Results:**

Immersion in 40 °C tap water caused *T*_ac_ to rise 0.64 ± 0.25 °C in the tap water session and 0.62 ± 0.27 °C in the CO_2_-enriched water session (*P* > 0.05). During the 30 °C water immersion, *T*_ac_ declined to the baseline within 13 ± 6 min in tap water and 10 ± 6 min in CO_2_-enriched water (*P* > 0.05). Cooling rates were 0.08 ± 0.06 °C/min in tap water and 0.08 ± 0.04 °C/min in CO_2_-enriched water (*P* > 0.05).

**Conclusions:**

CO_2_-enriched water containing 500 ppm CO_2_ did not cool faster than tap water immersion. This suggests that when the water temperature is 30 °C, a CO_2_ concentration of 500 ppm is insufficient to obtain the advantageous cooling effect during water immersion after body temperature has been increased.

## Background

It is well documented that immersion in CO_2_-enriched water causes cutaneous vasodilation at the immersed body surface [1–7]. This facilitates heat transfer from the body to the water, or from the water to the body. Applying this phenomenon, an earlier study compared the cooling effect of whole-body immersion in CO_2_-rich (1000 ppm) water with immersion in tap water after passive heating [8]. In that study, it was observed that immersion in CO_2_-rich water reduced ear canal temperature (*T*_ac_) about 1.7 times faster than tap water immersion. Similarly, Tanaka et al. [7] evaluated the cooling effect of immersing the forearms in cool water and reported that the decrease in ear canal temperature was slightly greater in CO_2_-rich water than in tap water. In both of those earlier studies, the CO_2_ concentration was set at 1000 ppm because that concentration is the lowest found in therapeutic springs [5].

However, there are problems associated with preparing artificial CO_2_-enriched water. For example, it takes time to dissolve CO_2_ in tap water. With the device used in previous studies, it takes > 20 min to prepare a full bathtub (about 200 L) of 1000 ppm CO_2_-rich water [2, 8]. In addition, the CO_2_ concentration gradually decreases over time. Considering actual usage, therefore, it is likely the CO_2_ concentration in the CO_2_-enriched water was lower than 1000 ppm, at least part of the time. For that reason, it is important to clarify whether a lower concentration (< 1000 ppm) of CO_2_ in the CO_2_-enriched water immersion can cool a body faster than tap water immersion. Schnizer et al. [6] previously examined the effect of CO_2_-enriched water on skin blood flow at water temperatures ranging from 22 to 38 °C and CO_2_ concentrations ranging from 0 to 4000 ppm. They showed that the amount of increase in skin blood flow depended on the CO_2_ concentration and suggested that the minimal effective concentration is 400–600 ppm. However, it is unknown whether water enriched with 400–600 ppm CO_2_ can actually cool the body faster than tap water after the body temperature has been increased. Therefore, to better understand the concentration dependency of the cooling effect of CO_2_-enriched water, the present study compared the cooling rates between water enriched with a lower concentration of CO_2_ and tap water during whole body water immersion after passive heating.

## Material and methods

### Participants

Ten healthy males (mean age = 20 ± 1 (SD) years; height = 168.0 ± 4.7 cm; weight = 61.4 ± 7.9 kg) participated in the study. The participants were all nonsmokers, and none were taking any medication. The study was approved by the research ethics committee of the University of Shizuoka (#1-24) and conformed to the provisions of the Declaration of Helsinki. Written informed consent was obtained from all participants.

### Experimental design

**Fig. 1:**
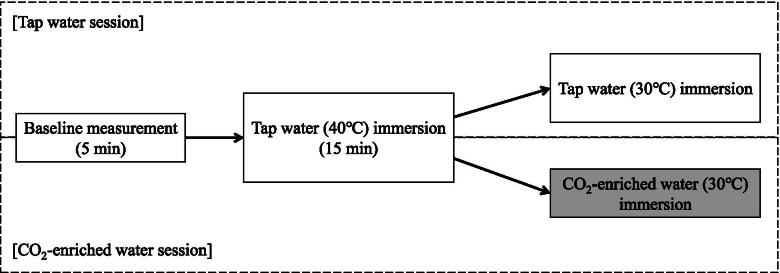
The experiment protocol. The participants initially sat in a chair for 5 min. During that time, baseline values were measured for each parameter. After the baseline measurements, the participants moved to a bath and were immersed in tap water at 40 °C for 15 min. The participants then moved to another bath and were immersed in tap water or CO_2_-enriched water at 30 °C

Each participant completed two sessions (immersion in tap water and in CO_2_-enriched water) within a 2-week period in random order. The participants were all asked to abstain from strenuous exercise and from consumption of alcohol during the 24 h before the experiment. In addition, all participants ate the same meal the night before the experiment and for breakfast on the day of the experiment. The experiment was conducted in the morning. After each participant came to the laboratory, they voided urine, were weighed, put on swimwear, and sat in a chair to rest. During this period, a heart rate (HR) monitor and thermocouples for recording skin temperature were attached. Then, an infrared temperature sensor was inserted into the auditory canal to record the temperature (*T*_ac_). During the experiments, *T*_ac_ data was collected using an infrared temperature sensor (BL100, Techno Next, Chiba, Japan), which was sampled every 1 s and averaged over 30-s periods. Skin temperature data were collected using copper constantan thermocouples, which were sampled every 1 s using a data logger system (DL350, Yokogawa, Tokyo, Japan) and averaged over 30-s periods. Skin temperatures were collected at four sites (chest, upper arm, thigh, and calf) and used to calculate the weighted mean skin temperature ($\overline{\mathrm{T}}$_sk_) [9]. HR was recorded every 5 s using a HR monitor (S810i, Polar, Finland) and averaged over 30-s periods. Figure 1 shows the experimental protocol. While the participants continued to sit in the chair, baseline values for each parameter were measured for 5 min. Once the baseline measurements were complete, the participants moved to a bath and were immersed to the axilla in tap water at 40 °C for 15 min to raise their body temperature. The participants then moved to another bath and were immersed to the axilla in tap water or CO_2_-enriched water at 30 °C to reduce body temperature. A water temperature of 30 °C was selected to avoid causing shivering or discomfort. Thermal comfort and thermal sensation were recorded every 5 min while the participant was immersed in the 30 °C tap or CO_2_-enriched water. Thermal comfort was evaluated using a four-point scale (1: comfortable—4: very uncomfortable), while thermal sensation was evaluated using a seven-point scale (1: cold—7: hot) [10]. Because bubbles attach to the body during immersion in CO_2_-enriched water, the water (both tap water and CO_2_-enriched water) was stirred at 13 L/min to keep the participants unaware of which water they were immersed in. The experiments terminated when any of the following were occurred: (1) *T*_ac_ dropped to baseline level, (2) the participants asked to terminate the experiment, or (3) *T*_ac_ did not change after more than 3 min. The CO_2_-enriched water was prepared using a device designed to dissolve CO_2_ in tap water (SC401, Mitsubishi Chemical Aqua Solutions, Tokyo, Japan) [2, 8]. In previous studies, where the water was enriched with 1000 ppm CO_2_ [2, 3, 5, 6, 8], it took > 20 min to prepare a full bathtub (about 200 L) of CO_2_-enriched water [2, 8]. The present study aimed to prepare 400–600 ppm CO_2_-enriched water within < 10 min. As a result, the CO_2_ concentration was maintained at about 500 ppm throughout the experiment. The experiments were carried out in a laboratory maintained at 23–25 °C and 40–60% relative humidity.

### Statistical analysis

All values are reported as means ± SD. Statistical analyses were performed using IBM SPSS Statistics (version 27, IBM Corp., NY, USA). Two-way ANOVA with repeated measures was conducted using time (levels: 1, 2, 3, 4, 5, 6, 7, 8, and 9 min during immersion in 30 °C tap or CO_2_-enriched water) and condition (levels: tap and CO_2_-enriched water) as factors. Times at which the numbers of participants were reduced (≥ 10 min) were not analyzed. Paired *t* tests were used to compare the tap water and CO_2_-enriched water sessions with respect to the changes in *T*_ac_ from that reached during the immersion in 40 °C tap water, the cooling times, cooling rates (slope of the regression line between *T*_ac_ and cooling time), thermal comfort, and thermal sensation. Values of *P* < 0.05 were considered significant.

## Results

**Fig. 2:**
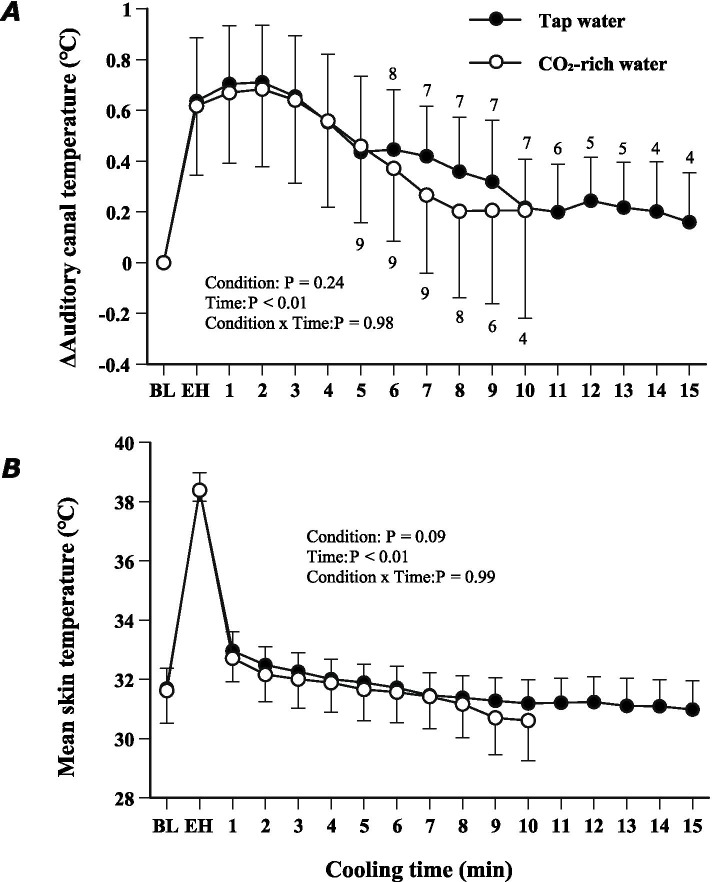
Time-dependent changes in change in auditory canal temperature (**A**) and mean skin temperature (**B**). The numbers adjacent to the symbols in (**A**) indicate the numbers of participants still immersed at the corresponding time; the numbers in (**A**) also apply to (**B**). Condition and time are the two factors considered in the ANOVA; condition × time is their interaction. *BL* baseline, *EH* end of heating

At baseline, *T*_ac_ was 36.0 ± 0.5 °C in the tap water condition and 35.8 ± 0.6 °C in the CO_2_-enriched water condition. Figure 2 shows the time-dependent changes in *T*_ac_. After immersion for 15 min in the 40 °C tap water bath, *T*_ac_ had risen by 0.64 ± 0.25 °C in the tap water condition and by 0.62 ± 0.27 °C in the CO_2_-enriched water condition (*P* > 0.05) (Fig. 2A). After subsequent immersion in the 30 °C bath, the time required for *T*_ac_ to return to baseline was 13 ± 6 min in the tap water condition and 10 ± 6 min in the CO_2_-enriched water condition (*P* > 0.05). The cooling rates were 0.08 ± 0.06 °C/min in the tap water condition and 0.08 ± 0.04 °C/min in the CO_2_-enriched water condition (*P* > 0.05). *T*_ac_ did not return to the baseline level in three participants in the tap water condition and in one participant in the CO_2_-enriched water condition. During the cooling, there was a significant main effect of cooling time (*F* = 6.37, *P* < 0.01). However, there was no significant main effect of condition (*F* = 1.41, *P* = 0.24) and no interaction between the condition and cooling time (*F* = 0.23, *P* = 0.98).

Before heating, $\overline{\mathrm{T}}$_sk_ was 31.7 ± 0.7 °C in the tap water condition and 31.6 ± 1.1 °C in the CO_2_-enriched water condition. After immersion for 15 min in the heated bath, $\overline{\mathrm{T}}$_sk_ had risen to 38.4 ± 0.6 °C in tap water and to 38.4 ± 0.4 °C in CO_2_-enriched water. $\overline{\mathrm{T}}$_sk_ rapidly declined during cooling, and there was a significant main effect of cooling time (*F* = 7.13, *P* < 0.01), but there was no significant main effect of condition (*F* = 2.88, *P* = 0.09) and no interaction between the condition and cooling time (*F* = 0.11, *P* = 0.99) (Fig. 2B).

**Fig. 3:**
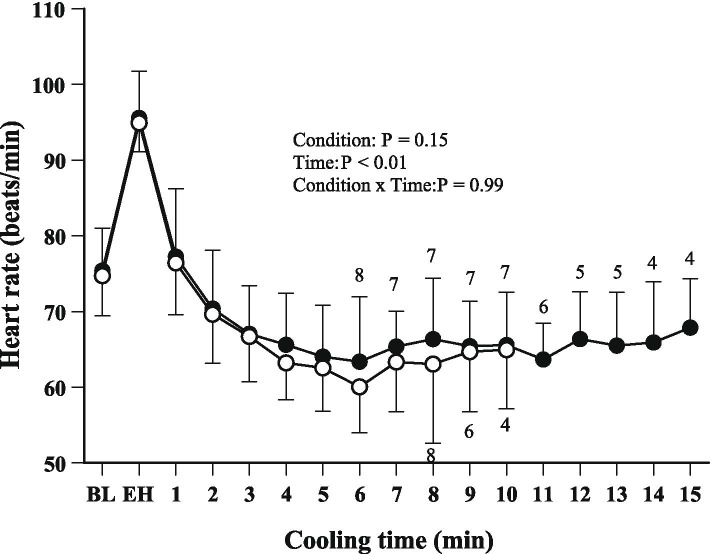
Time-dependent changes in heart rate. Because heart rate could not be measured in one subject, the data presented are from the remaining nine subjects. The numbers adjacent to the symbols indicate the numbers of participants still immersed at the corresponding time. Condition and time are the two factors considered in the ANOVA; condition × time is their interaction. *BL* baseline, *EH* end of heating

Figure 3 shows the time-dependent changes in HR. Before heating, HR was 75 ± 6 beats/min in the tap water condition and 75 ± 5 beats/min in the CO_2_-enriched water condition. By the end of heating, HR had increased to 96 ± 6 beats/min in the tap water condition and to 95 ± 4 beats/min in the CO_2_-enriched water condition. HR decreased during cooling in both conditions, and there was a significant main effect of cooling time (*F* = 7.29, *P* < 0.01). On the other hand, there was no significant main effect of condition (*F* = 2.07, *P* = 0.15) or interaction between condition and cooling time (*F* = 0.10, *P* = 0.99).

Table 1 shows the thermal comfort and sensation reported during cooling. There was no significant between-condition difference in thermal comfort or thermal sensation after 5 min of cooling.

**Table 1:** Thermal comfort and sensation during cooling

	5 min	10 min	15 min
Thermal comfort			
Tap water	1.5 ± 0.5	2.0 ± 0.8 (*n* = 7)	2.3 ± 1.0 (*n* = 4)
CO_2_-enriched water	1.6 ± 0.7	2.0 ± 0.8 (*n* = 4)	–
Thermal sensation			
Tap water	2.9 ± 0.9	2.4 ± 1.4 (*n* = 7)	2.5 ± 1.3 (*n* = 4)
CO_2_-enriched water	2.9 ± 1.0	3.8 ± 1.3 (*n* = 4)	–

Thermal comfort scale (1: comfortable—4: very uncomfortable). Thermal sensation scale (1: cold—7: hot). Values are means ± SD

## Conclusions

The present study shows that CO_2_-enriched water containing 500 ppm CO_2_ does not facilitate heat transfer from the body to the water. It was previously reported that immersion in CO_2_-enriched water containing 1000 ppm CO_2_ cooled the body 1.7 times faster than immersion in tap water [8]. That was not the case with the lower concentration of CO_2_ used in the present study. Schnizer et al. [6] examined the effect of CO_2_-enriched water on skin blood flow at different water temperatures (22–38 °C) and CO_2_ concentrations (0–4000 ppm). They reported that the magnitude of the increase in skin blood flow depended on the CO_2_ concentration and the water temperature and that the latency of the increase in skin blood flow increased with decreases in water temperature. In addition, Ito et al. [1] examined the effect of water temperature and CO_2_ concentration on skin blood flow in rats while dissolving CO_2_ into the freshwater bath during a 20-min water immersion. They reported that skin blood flow gradually increased during the CO_2_-enriched water immersion and that skin blood flow increased with increases in the dissolved CO_2_ concentration, even at a water temperature of 23 °C. These results suggest that a CO_2_ concentration of 500 ppm in water was insufficient to facilitate heat transfer from the body during a 10-min water immersion. Moreover, there was no significant between-condition difference in thermal comfort or thermal sensation, indicating that immersion in water containing a low CO_2_ concentration after passive heating does not alleviate the sensation of discomfort. Previous studies reported that immersion in CO_2_-enriched water produced a warmer, more comfortable sensation than immersion in fresh water [2, 5, 8]. Given the present observations that there were no significant differences in Δ*T*_ac_, thermal comfort, or thermal sensation, it is suggested that, at 500 ppm, there is insufficient diffusion of CO_2_ into cutaneous blood vessels to exert a beneficial effect.

On the other hand, Sato et al. [4] reported that when participants were immersed in a hot bath, even 100 ppm CO_2_ enhanced skin blood flow and sweating measured at the chest as compared to freshwater immersion. The difference between the present study and Sato’s study is water temperature. In their study, measurements of skin blood flow were made at a water temperature of 40 °C. During immersion in hot water, even fresh water, skin blood flow is augmented [4]. Although skin blood flow was not measured in the present study, the water temperature was set at 30 °C, and $\overline{\mathrm{T}}$_sk_ was always lower than 34 °C during immersion. This suggests that the degree of cutaneous vasodilation was almost certainly smaller than in Sato’s study. Previous studies [1, 6] reported that the magnitude of the increase in skin blood flow during CO_2_-enriched water immersion was dependent on the water temperature. It is therefore possible that the amount of CO_2_ diffusing into the cutaneous blood vessels was higher in those earlier studies than in the present study.

In summary, the results of the present study suggest that immersion in water containing a low concentration of CO_2_ (500 ppm) does not cool the body faster than immersion in tap water after passive heating, nor does it alleviate the sensation of discomfort when the water temperature is set at 30 °C. These observations suggest that a higher CO_2_ concentration is necessary to obtain the advantageous cooling effect and alleviate discomfort during water immersion after hyperthermia. For practical application, the present results suggest that a high concentration of CO_2_ in water is necessary to enhance body temperature reduction, even if it takes time to prepare, and that it is necessary to maintain that high CO_2_ concentration throughout the immersion period.

## Data Availability

The datasets used and/or analyzed during this study are available from the corresponding author on reasonable request.

## Abbreviations

ANOVA: Analysis of variance
CO_2_: Carbon dioxide
HR: Heart rate
*T*_ac_: Auditory canal temperature
$\overline{\mathrm{T}}$_sk_: Mean skin temperature
